# Association of a Lifestyle Risk Index With Visceral and Subcutaneous Adipose Tissue in the German National Cohort (NAKO)

**DOI:** 10.1002/oby.70071

**Published:** 2025-11-19

**Authors:** Gertraud Maskarinec, Rebecca Klapp, Ute Nöthlings, Matthias B. Schulze, Fabian Bamberg, Jürgen Machann, Sabrina Schlesinger, Michael Leitzmann, Anja Sedlmeier, Patricia Bohmann, Susanne Rospleszcz, Johanna Nattenmüller, Tobias Haueise, Karen Steindorf, Thoralf Niendorf, Christopher L. Schlett, Karin Halina Greiser, Leo Panreck, Jakob Linseisen, Christina‐Alexandra Conzen, Sylvia Gastell, Tamara Schikowski, Henry Völzke, Robin Bülow, Annette Peters, Fiona Niedermayer, Rudolf Kaaks, Heiko Becher, André Karch, Klaus Berger, Thomas Keil, Lilian Krist, Michael Hoffmeister, Ute Mons, Boerge Schmidt, Andreas Stang, Rafael Mikolajczyk, Alexander Kluttig, Wolfgang Lieb, Cara Övermöhle, Antje Hebestreit, Kathrin Günther, Volker Harth, Nadia Obi, Stefanie Castell, Robyn Kettlitz, Katharina Nimptsch, Tobias Pischon

**Affiliations:** ^1^ University of Hawaii Cancer Center Honolulu Hawaii USA; ^2^ Max‐Delbrück Center for Molecular Medicine in the Helmholtz Association Berlin Germany; ^3^ Institute of Nutritional and Food Sciences‐Nutritional Epidemiology University of Bonn Bonn Germany; ^4^ German Institute of Human Nutrition Potsdam‐Rehbrücke Nuthetal Germany; ^5^ Institute of Nutritional Science University of Potsdam Nuthetal Germany; ^6^ Department of Diagnostic and Interventional Radiology, Medical Center – University of Freiburg, Faculty of Medicine University of Freiburg Freiburg Germany; ^7^ Institute for Diabetes Research and Metabolic Diseases Helmholtz Munich at the University of Tübingen Tübingen Germany; ^8^ German Center for Diabetes Research (DZD) Tübingen Germany; ^9^ Institute for Biometrics and Epidemiology, German Diabetes Center Leibniz Center for Diabetes Research at the Heinrich Heine University Düsseldorf Germany; ^10^ Institute for Epidemiology and Preventive Medicine University of Regensburg Regensburg Germany; ^11^ Institute for Radiology and Nuclear Medicine Hirslanden Clinic St. Anna Luzern Switzerland; ^12^ Division of Physical Activity, Prevention and Cancer, German Cancer Research Center (DKFZ) Heidelberg Germany; ^13^ Division of Cancer Epidemiology, German Cancer Research Center (DKFZ) Heidelberg Germany; ^14^ NAKO e.V. Heidelberg Germany; ^15^ Epidemiology, Medical Faculty University of Augsburg Augsburg Germany; ^16^ IUF‐Leibniz Research Institute for Environmental Medicine Düsseldorf Germany; ^17^ Institute for Community Medicine University Medicine Greifswald Greifswald Germany; ^18^ Institute of Epidemiology Helmholtz Zentrum München ‐ German Research Center for Environmental Health (GmbH) Neuherberg Germany; ^19^ German Center for Diabetes Research (DZD e.V.) Neuherberg Germany; ^20^ Institute for Medical Information Processing, Biometry, and Epidemiology Faculty of Medicine, LMU Munich München Germany; ^21^ Institute for Global Health University Hospital Heidelberg Heidelberg Germany; ^22^ Institute of Epidemiology and Social Medicine University of Münster Münster Germany; ^23^ Institute of Social Medicine, Epidemiology and Health Economics Charité – Universitätsmedizin Berlin Berlin Germany; ^24^ Institute of Clinical Epidemiology and Biometry University of Würzburg Würzburg Germany; ^25^ State Institute of Health I, Bavarian Health and Food Safety Authority Erlangen Germany; ^26^ Division of Clinical Epidemiology and Aging Research German Cancer Research Center (DKFZ) Heidelberg Germany; ^27^ Division of Primary Cancer Prevention German Cancer Research Center (DKFZ) Heidelberg Germany; ^28^ Institute for Medical Informatics, Biometry and Epidemiology, University Hospital Essen University of Duisburg‐Essen Essen Germany; ^29^ Institute for Medical Epidemiology, Biometrics and Informatics Martin Luther University Halle‐Wittenberg Halle (Saale) Germany; ^30^ Institute of Epidemiology Kiel University Kiel Germany; ^31^ Leibniz Institute for Prevention Research and Epidemiology – BIPS Bremen Germany; ^32^ Institute for Occupational and Maritime Medicine (ZfAM) University Medical Center Hamburg‐Eppendorf Hamburg Germany; ^33^ Department for Epidemiology, Helmholtz Centre for Infection Research Brunswick Lower Saxony Germany

**Keywords:** body fat distribution, cohort study, lifestyle behaviors, obesity

## Abstract

**Objective:**

This cross‐sectional study examined a Lifestyle Risk Factor Index (LSRI) in relation to adiposity measures including visceral adipose tissue (VAT) in the German National Cohort (NAKO).

**Methods:**

Based on self‐reports at baseline among 30,920 of > 205,000 NAKO eligible participants with magnetic resonance imaging (MRI) scans, one point each for not smoking, adhering to ≥ 3/7 diet recommendations, consuming ≤ 1 (women)/≤ 2 (men) alcoholic drinks/day, and ≥ 150 min/week physical activity was assigned. VAT volume, obtained from whole‐body MRI at 3T, was analyzed by deep learning‐based image segmentation. General linear models estimated adjusted geometric mean adiposity measures by LSRI and stratified analyses by sex and BMI.

**Results:**

Of 18,508 participants aged 48.2 ± 12.2 years, the respective proportions for 0/1, 2, 3, and 4 LSRI points were 7%, 24%, 51%, and 18%. Participants with LSRI scores of 4 versus 0/1 had lower adjusted geometric mean volumes of VAT (2.3; 95% CI 2.2, 2.3 vs. 3.0; 95% CI 2.9, 3.1 L). These differences were slightly attenuated after adding BMI. This association was weaker for individuals with obesity than normal/overweight.

**Conclusion:**

A combination of lifestyle factors appears to be associated with lower VAT volume, but an elevated BMI may have a greater influence on VAT accumulation than lifestyle behaviors alone.

**Study Importance**

- What is already known?
  ○ Lifestyle risk factors are important predictors of body weight and chronic diseases.
- What does this study add?
  ○ Based on a large study population, this analysis shows the significance of lifestyle behaviors on body fat distribution, specifically visceral adipose tissue, which appears to have adverse metabolic effects.

## 1 Introduction

Common lifestyle behaviors may affect body fat distribution, in particular visceral adipose tissue (VAT) accumulation. VAT is mainly located in the mesentery and omentum and, therefore, has direct proximity to the liver. It contains a large number of inflammatory immune cells and a greater percentage of large adipocytes, which make VAT more metabolically active than subcutaneous adipose tissue (SAT) [1, 2]. Evidence for the hypothesis that VAT is a predictor of morbidity and mortality beyond body mass index (BMI) [3, 4, 5] comes from studies that found waist circumference (WC) used as a surrogate of VAT mass was more strongly related to the risk of type 2 diabetes (T2D), cardiovascular disease, certain cancers, and mortality than BMI [6]. In addition, the stronger association of BMI with T2D [7, 8] and breast cancer [9, 10] in persons with Asian ancestry who accumulate a higher proportion of VAT than other ethnic groups supports the importance of VAT [8]. The growing literature based on imaging‐based measures has reported that men accumulate more VAT than women while SAT values are substantially greater in women than men [11, 12]. Also, a higher range of variability in VAT and SAT was reported for participants with obesity compared to individuals with normal weight [12].

Considerable evidence for a protective role of lifestyle behaviors against VAT accumulation is available. These include avoidance of smoking [13], a high‐quality diet, for example, as assessed by the Healthy Eating Index or a Mediterranean Diet Score [14], low‐to‐moderate alcohol intake [15, 16], and adequate physical activity [17, 18], which are all related to a more favorable body fat distribution. Different lifestyle behaviors have also been investigated in combination. An association of a composite measure, the Lifestyle Risk Factor Index (LSRI), with body fat distribution was recently shown in the Multiethnic Cohort [19]. This LSRI is thought to capture the overall benefit of four behaviors and ranges from 0 to 4 points, where a higher score indicates a more favorable lifestyle [20]. All four components as well as a combination of risk factors have been associated not only with body fat distribution but also with reduced chronic disease incidence and mortality [21, 22, 23]. Given that the different behavioral risk factors that influence VAT accumulation often occur together and are highly correlated, it is of interest to evaluate their relation to body fat distribution in combination beyond their individual impact. Based on the hypothesis that these lifestyle risk factors affect body fat distribution, the goal of the current analysis is to understand the combined and individual associations of lifestyle factors with different body fat components measured at the baseline examination of the German National Cohort (NAKO‐Gesundheitsstudie, NAKO), a large population‐based study with magnetic resonance imaging (MRI)‐based body fat assessment [24]. Therefore, our objective was to determine the relationship of a composite LSRI that captures the interaction of four health behaviors with VAT and, for comparison, with four additional adiposity measures, that is, BMI, WC, SAT, and the VAT/SAT ratio, using data from the NAKO. In addition, we aimed to examine this association for effect modification by sex and/or BMI.

## 2 Methods

### 2.1 Study Sample

**FIGURE 1:**
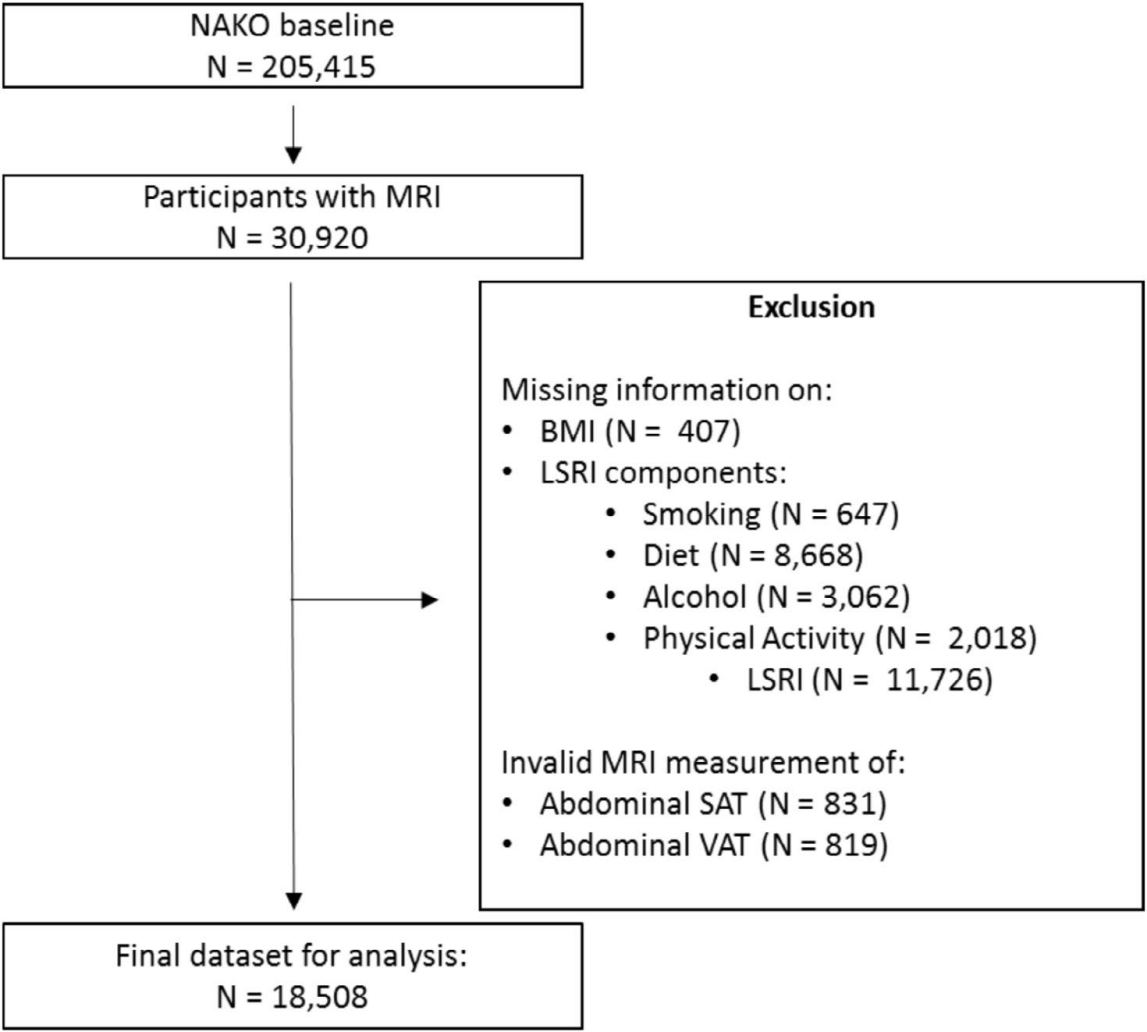
Flowchart for the NAKO study population in the current analysis. LSRI, Lifestyle Risk Factor Index; MRI, magnetic resonance imaging; NAKO, German National Cohort; SAT, subcutaneous adipose tissue; VAT, visceral adipose tissue.

The current project used a cross‐sectional study design to analyze VAT and SAT as derived from MRI in relation to LSRI. The NAKO is a prospective cohort study with more than 205,000 participants aged 20–69 years at baseline [24]. Recruitment was performed at 18 study centers throughout Germany based on random sampling through population registries. Overall, 30,920 participants from 11 study centers who had no contraindication underwent full‐body MRI scanning at five MRI centers (Figure 1) [25]. Of these, 18,508 with complete information on LSRI and adiposity measures under investigation were included in the present analysis after excluding cohort members (some overlap) with missing values for VAT (*N* = 819), SAT (*N* = 831), BMI (*N* = 407), smoking (*N* = 647), diet (*N* = 8668), alcohol consumption (*N* = 3062), and physical activity (*N* = 2018). Based on ANOVA and chi‐square tests, the excluded participants did not differ by sex (*p* = 0.26), but they were less likely to be current smokers (*p* < 0.0001) and had higher BMI (+0.83 kg/m^2^, *p* < 0.0001) and VAT volume (+0.26 L, *p* < 0.0001). All study documents, including study protocols, participant information documents, and declaration of consent forms for the baseline examinations including the MRI scans, were approved by relevant ethical committees [24].

### 2.2 Data Collection

As part of the initial study visit (baseline) in 2014–2019, participants completed touch screen questionnaires and face‐to‐face interviews about demographic information, smoking, alcohol intake, medical history, and other health‐related information. The question about smoking referred to tobacco products only; other substances were not included. A Food Frequency Questionnaire (FFQ), which was developed as a web application and a print version, allowed estimation of dietary intakes [26] and was based on the German version of the multilingual European Food Propensity Questionnaire [27]. Four of the authors identified FFQ items that were relevant to the seven food groups, that is, fruits including juice (not sugared drinks), vegetables including potatoes, fish, processed meats, unprocessed red meats, whole grains, and refined grains, and computed total frequency per group. Physical activity was assessed using the Global Physical Activity Questionnaire (GPAQ) asking about the average duration of physical activity in minutes per week without taking intensity into account [28]. Anthropometric measures including WC were assessed by trained personnel during the study center visit. BMI categories (normal weight: < 25; overweight: 25–< 30; and obesity: ≥ 30 kg/m^2^) were assigned using measured weight and height [24].

### 2.3 MRI

As part of the baseline exam, whole‐body MRI was performed by 3T imaging at five dedicated imaging sites following similar protocols, some of which also examined participants from other study centers [25]. As outcomes for the current analysis, the VAT and SAT volumes in liters were obtained from MRI based on a T1‐weighted 3D two‐point DIXON MRI sequence in axial orientation (3 mm slice thickness, 1.4 × 1.4 mm in‐plane voxel size). For quality assurance, a central MRI core served as a central reference, the study personnel was centrally trained, the MRI protocol was identical at all sites, and local board‐certified radiologists oversaw the program [25]. The respective volumes of the abdominal scans were analyzed by deep learning‐based image segmentation [12].

### 2.4 LSRI

As exposure, a score of lifestyle risk factors, which we adopted from a previous publication based on the UK Biobank [20], was computed from data collected at the NAKO baseline exam. For each component, one point was given if the participant met the definition, which resulted in a range of 0 to 4 points. The points were assigned as follows (Table S1): one point each for current non‐smoking as reported at the baseline exam, adhering to ≥ 3/7 dietary recommendations, consuming ≤ 1 (women)/≤ 2 (men) alcoholic drinks/day (1 drink equals 14 g alcohol), and performing ≥ 150 min/week physical activity. The criterion for diet was reduced from scoring 4/7 food groups as proposed by Lourida et al. [20] to 3/7 food groups as adherence to the diet guideline was quite low among NAKO participants. Given the small number of individuals with a total score of 0 points, they were combined with those who obtained 1 point.

### 2.5 Statistical Analysis

Descriptive statistics were generated for demographic, anthropometric, and lifestyle variables. Due to their non‐normal distributions, geometric means and 95% confidence intervals (95% CI) were computed for VAT, SAT, and the VAT/SAT ratio. To estimate the association between the LSRI and anthropometric measures, we applied general linear models with adjustment for sex, age (continuous), and in some models also BMI (continuous) to obtain regression coefficients and adjusted means. The linear models computed mean BMI (kg/m^2^), WC (cm), VAT and SAT volumes (L), and VAT/SAT ratio by LSRI category as well as a trend test using the continuous LSRI variable. In a sensitivity analysis, the 143 participants with underweight (< 18.5 kg/m^2^) were excluded to assess possible error. For the three MRI‐based parameters, adjusted models were applied using log‐transformed variables and subsequently back‐transformed. The geometric means (for VAT, SAT, and VAT/SAT ratio) and arithmetic means (for WC) of the unadjusted measures were compared to two adjusted models, one with age and sex as covariates and the second one with BMI added to examine the influence of abdominal adiposity independent of overall adiposity. These two models were also applied to the four individual LSRI components. Standardized variables of the adiposity measures or their logarithm were modeled to compare the respective strengths of their associations with LSRI. Given VAT's association with BMI, its well‐known differences by sex, and the stronger correlations of VAT with BMI reported in women than men [12], we explored the possibility of effect modification using stratified models by sex and BMI. All statistical analyses were conducted using the statistical software package SAS Enterprise Guide 8.4 Update 2 (SAS Institute Inc., Cary, NC, USA).

## 3 Results

Of 18,508 participants with complete information (Table 1), 43% of the participants were classified as normal weight, 38% as overweight, and 18% as having obesity based on BMI. The geometric mean for VAT was 2.70 L (95% CI 2.67, 2.73 L; Q1‐Q3: 1.60–4.75 L) with higher geometric mean volumes for men (3.71 L, 95% CI 3.66, 3.75 L; Q1‐Q3: 2.49–5.85 L) than for women (1.81 L, 95% CI 1.78, 1.83 L; Q1‐Q3: 1.11–2.99 L). For SAT, the geometric mean was 5.80 L (95% CI 5.57, 5.84 L; Q1‐Q3: 4.16–8.18 L) with higher volumes for women (6.48 L, 95% CI 6.41, 6.55 L; Q1‐Q3: 4.58–11.17 L) than for men (5.31 L, 95% CI 5.26, 5.36 L; Q1‐Q3: 3.88–7.43 L).

**TABLE 1:** Characteristics of the NAKO study sample by LSRI score.

Characteristic	Group	All, *N* (%)	LSRI score			
			0/1	2	3	4
*N*		18,508	1210	4532	9463	3303
%		100	7	24	51	18
Lifestyle Risk Factor Index components						
Current smoking, *N* (%)	Yes	3128 (17)	31	55	14	0
	No	15,380 (83)	2	18	59	21
Healthy diet, food recommendations, *N* (%)	< 3 of 7	13,823 (75)	9	31	60	0
	≥ 3 of 7	4685 (25)	0	4	25	71
Alcohol intake, drinks/day, *N* (%)	> 1 or 2	2653 (14)	29	53	18	0
	≤ 1 or 2	15,855 (86)	3	20	57	21
Physical activity, min/week, *N* (%)	< 150	2661 (14)	30	60	11	0
	≥ 150	15,847 (86)	3	19	58	21
Demographics						
Sex, *N* (%)	Male	10,311 (56)	7	26	54	13
	Female	8197 (44)	6	23	47	24
Age, years, mean ± SD		48.2 ± 12.2	48.3 ± 10.8	48.5 ± 11.6	47.9 ± 12.3	48.7 ± 12.7
Anthropometrics						
BMI, kg/m^2^, *N* (%)	< 18.5	143 (1)	11	19	49	21
	18.5–< 25	7965 (43)	6	22	51	21
	25–< 30	7090 (38)	7	26	51	16
	≥ 30	3310 (18)	7	27	51	14
BMI, kg/m^2^, mean ± SD		26.2 ± 4.5	26.7 ± 4.6	26.6 ± 4.6	26.3 ± 4.5	25.5 ± 4.4
WC, cm, mean ± SD^a^		90.1 ± 13.3	92.9 ± 13.8	91.8 ± 13.5	90.3 ± 13.2	86.5 ± 12.5
Abdominal VAT, L,		2.70	3.20	3.01	2.72	2.13
geometric mean (95% CI)		(2.67, 2.73)	(3.08, 3.32)	(2.95, 3.07)	(2.68, 2.76)	(2.08, 2.19)
Abdominal SAT, L,		5.80	6.07	6.05	5.74	5.53
geometric mean (95% CI)		(5.75, 5.84)	(5.89, 6.24)	(5.96, 6.14)	(5.68, 5.80)	(5.43, 5.63)
Abdominal VAT/SAT ratio,		0.47	0.53	0.50	0.47	0.39
geometric mean (95% CI)		(0.46, 0.47)	(0.51, 0.54)	(0.49, 0.51)	(0.47, 0.48)	(0.38, 0.39)

Abbreviations: LSRI, Lifestyle Risk Factor Index; SAT, subcutaneous adipose tissue; VAT, visceral adipose tissue; WC, waist circumference.

^a^ *N* = 7 has missing information.

The proportions of participants with LSRI scores of 0/1, 2, 3, and 4 were 7%, 24%, 51%, and 18%, respectively (Table 1). As to individual LSRI components, 83% of participants received a point for not currently smoking, 25% for a healthy diet, 86% for low‐to‐moderate alcohol consumption, and 86% for meeting the physical activity requirement. Adherence to food groups was highest for fruits including juice (39%), refined grains (38%), and vegetables including potatoes (33%), while it was low for unprocessed red meats (18%), fish (17%), processed meats (10%), and whole grains (2%). Women presented a more favorable pattern of LSRI scores than men, and participants with normal weight had a more favorable score than individuals with overweight or obesity. The LSRI was inversely related to all adiposity measures; the respective values for BMI, WC, VAT, SAT, and VAT/SAT were around 5%, 7%, 33%, 9%, and 26% lower in the highest versus lowest LSRI category (*p* < 0.0001).

**FIGURE 2:**
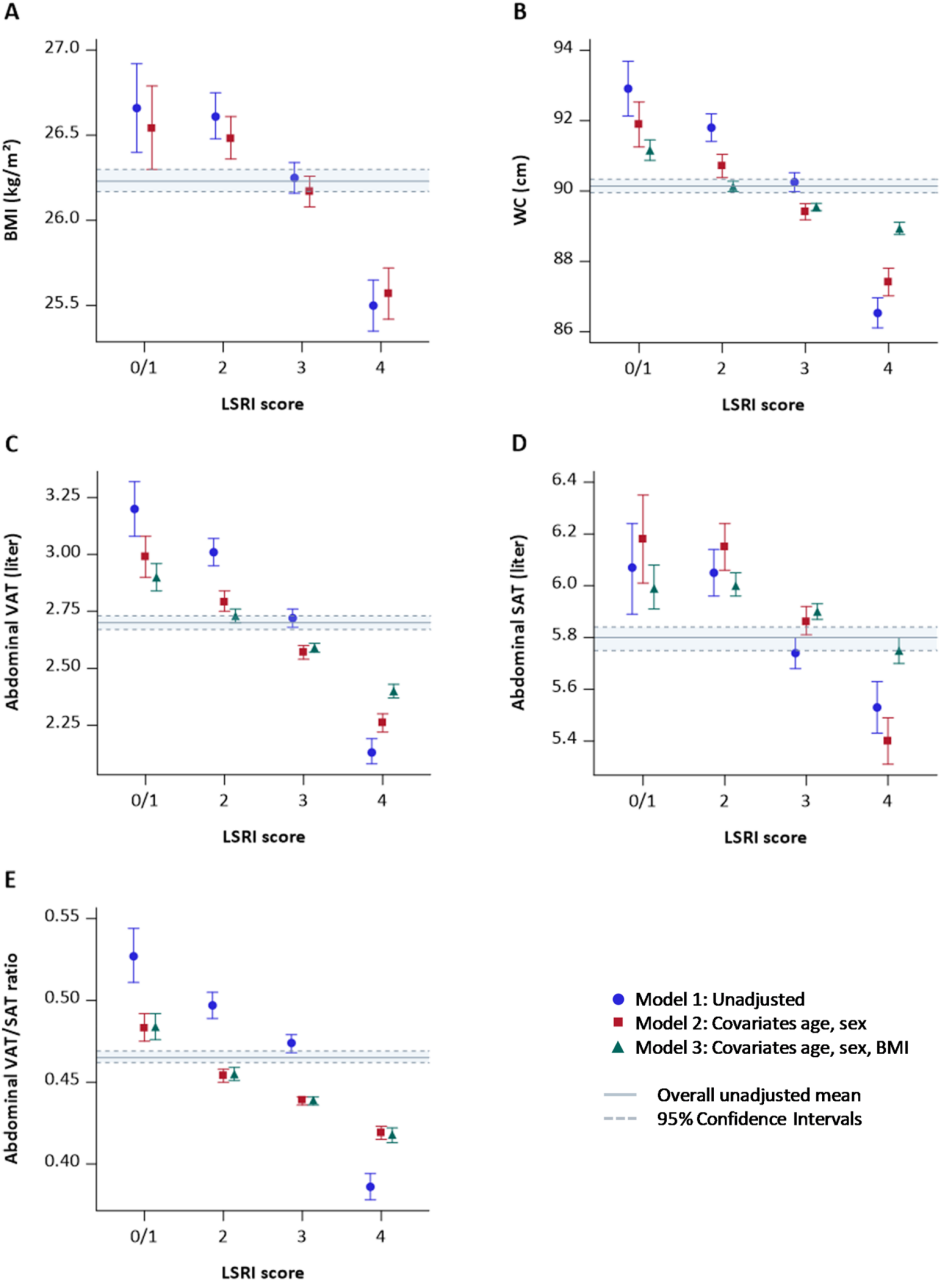
Geometric and arithmetic means of anthropometric measures by LSRI category. Adjusted and unadjusted arithmetic means (panels A, B) and geometric means (panels C, D, E) ± 95% CI obtained by general linear model with (A) BMI, (B) waist circumference (WC), (C) abdominal visceral adipose tissue (VAT), (D) abdominal subcutaneous adipose tissue (SAT), (E) abdominal VAT/SAT ratio as dependent variable and Lifestyle Risk Factor Index (LSRI) category (0/1–4 points) as independent variable with different covariates (Model 1–3) and compared to the overall unadjusted mean ±95% CI.

The unadjusted and age‐ and sex‐adjusted geometric (VAT, SAT, VAT/SAT ratio) and arithmetic (BMI, WC) means for all adiposity measures were lower with higher LSRI scores (Figure 2). The greatest difference was observed for those with a score of 4 LSRI points compared to a score of 0/1, although values for those with 3 points were also lower compared to scores of 0/1 and 2, as indicated by the 95% CI. Adding BMI to the model slightly attenuated but did not eliminate the associations with VAT, SAT, VAT/SAT, and WC. Comparing all five body fat or anthropometric measures, the percent difference between the highest and the lowest LSRI category in the age‐, sex‐, and BMI‐adjusted model was greatest for VAT (17%) and the VAT/SAT ratio (12.5%), while it was small for SAT (5%), BMI (3.7%), and WC (2.5%) (Table S2). For example, participants with a LSRI score of 4 versus 0/1 points had lower mean levels of VAT (2.3; 95% CI 2.2, 2.3 vs. 3.0; 95% CI 2.9, 3.1 L) in the age‐ and sex‐adjusted model, and that difference persisted after including BMI in the model (2.4; 95% CI 2.4, 2.4 vs. 2.6; 95% CI 2.8, 3.0 L). Exclusions of the 143 participants with underweight did not substantially change the findings (data not shown).

The stronger association between LSRI and VAT than the other four adiposity measures was confirmed in age‐ and sex‐adjusted models using standardized adiposity measures (Table S3). The standardized regression coefficients indicated lower values per 1‐point increase in LSRI, but the association was strongest for VAT (−0.13), followed by WC (−0.11) and SAT (−0.10), and weakest for BMI (−0.08) and the VAT/SAT ratio (−0.07). However, in BMI‐adjusted models, VAT and the VAT/SAT ratio showed equal strength in association with LSRI (−0.08), while the coefficients for WC (−0.05) and SAT (−0.03) were smaller.

As to individual LSRI components, all four lifestyle factors were inversely associated with VAT volume after including BMI as a covariate (Table 2). The differences in adjusted geometric means between adherence and non‐adherence in the BMI‐adjusted model were greater for VAT than SAT, with lower volumes for adhering to diet (VAT 7.1% and SAT 2.2%) and physical activity recommendations (VAT 10.1% and SAT 6.6%). In the case of smoking, current non‐smoking was associated with higher SAT values than current smoking. Current non‐smoking showed the smallest difference by adherence, with 1.5% lower VAT and 2.4% higher SAT among non‐smokers. For low‐to‐moderate alcohol consumption, adherence was associated with a nonsignificantly higher SAT volume (< 1%), whereas VAT volume was 5.0% lower. In contrast, relative differences for BMI and WC by adherence status to all four LSRI components were considerably smaller than for VAT (< 3%).

**TABLE 2:** Association of individual lifestyle factors with anthropometric measures.

LSRI component	Model	Group	Abdominal VAT (L)^a^			*p*	Abdominal SAT (L)^a^			*p*	Abdominal VAT/SAT ratio^a^			*p*
			Mean	95% CI										
Mean	95% CI	
Mean	95% CI	
Current smoking	1	Yes	2.72	2.66	2.78		5.86	5.74	5.98		0.46	0.46	0.47	
		No	2.70	2.66	2.74	0.35	6.03	5.95	6.11	0.003	0.45	0.44	0.45	< 0.0001
	2	Yes	2.74	2.70	2.78		5.90	5.84	5.96		0.46	0.46	0.47	
		No	2.70	2.67	2.73	0.03	6.04	5.99	6.08	< 0.0001	0.45	0.44	0.45	< 0.0001
Healthy diet (food recommendations)	1	< 3 of 7	2.90	2.86	2.95		6.22	6.13	6.30		0.47	0.46	0.47	
		≥ 3 of 7	2.53	2.48	2.59	< 0.0001	5.68	5.58	5.79	< 0.0001	0.45	0.44	0.45	< 0.0001
	2	< 3 of 7	2.82	2.79	2.85		6.03	5.99	6.08		0.47	0.46	0.47	
		≥ 3 of 7	2.62	2.59	2.66	< 0.0001	5.90	5.84	5.96	< 0.0001	0.44	0.44	0.45	< 0.0001
Alcohol intake (drinks/day)	1	> 1 or 2	2.76	2.70	2.82		5.89	5.77	6.02		0.47	0.46	0.47	
		≤ 1 or 2	2.66	2.62	2.70	0.001	6.00	5.92	6.08	0.096	0.44	0.44	0.45	< 0.0001
	2	> 1 or 2	2.79	2.75	2.83		5.96	5.90	6.03		0.47	0.46	0.47	
		≤ 1 or 2	2.65	2.63	2.68	< 0.0001	5.97	5.93	6.01	0.80	0.44	0.44	0.45	< 0.0001
Physical activity (min/week)	1	< 150	2.96	2.89	3.03		6.39	6.25	6.52		0.46	0.46	0.47	
		≥ 150	2.48	2.45	2.52	< 0.0001	5.53	5.46	5.60	< 0.0001	0.45	0.45	0.45	< 0.0001
	2	< 150	2.87	2.82	2.91		6.18	6.11	6.25		0.46	0.46	0.47	
		≥ 150	2.58	2.56	2.61	< 0.0001	5.77	5.73	5.80	< 0.0001	0.45	0.44	0.45	< 0.0001

LSRI component	Model	Group	BMI (kg/m^2^)^b^			*p*	WC (cm)^b^			*p*
			Mean	95% CI						
Mean	95% CI	
Current smoking	1	Yes	26.2	26.0	26.3		90.3	89.9	90.8	
		No	26.2	26.1	26.3	0.472	90.2	89.9	90.5	0.500
	2	Yes					90.5	90.3	90.7	
		No					90.2	90.1	90.4	0.004
Healthy diet (food recommendations)	1	< 3 of 7	26.5	26.4	26.7		91.3	91.0	91.7	
		≥ 3 of 7	25.8	25.7	26.0	< 0.0001	89.2	88.8	89.6	< 0.0001
	2	< 3 of 7					90.6	90.5	90.8	
		≥ 3 of 7					90.1	89.9	90.3	< 0.0001
Alcohol intake (drinks/day)	1	> 1 or 2	26.1	25.9	26.3		90.5	90.0	91.0	
		≤ 1 or 2	26.3	26.2	26.4	0.073	90.1	89.7	90.4	0.066
	2	> 1 or 2					90.8	90.6	91.0	
		≤ 1 or 2					90.0	89.8	90.1	< 0.0001
Physical activity (min/week)	1	< 150	26.6	26.4	26.8		91.8	91.3	92.2	
		≥ 150	25.8	25.7	25.9	< 0.0001	88.8	88.5	89.1	< 0.0001
	2	< 150					90.9	90.7	91.2	
		≥ 150					89.8	89.6	89.9	< 0.0001

Abbreviations: LSRI, Lifestyle Risk Factor Index; SAT, subcutaneous adipose tissue; VAT, visceral adipose tissue; WC, waist circumference.

^a^ Adjusted Geometric means ± 95% CI and significance with *p* value obtained by general linear model with MRI‐based adipose tissue measurement (VAT, SAT, VAT/SAT ratio) as dependent variable and LSRI components as independent variable. Model 1: all other LSRI components + sex, age, Model 2: Model 1 + BMI.

^b^ Adjusted Arithmetic means ± 95% CI and significance with *p* value obtained by general linear model with BMI or WC as dependent variable and LSRI components as independent variable. Model 1: all other LSRI components + sex, age; Model 2: Model 1 + BMI.

**FIGURE 3:**
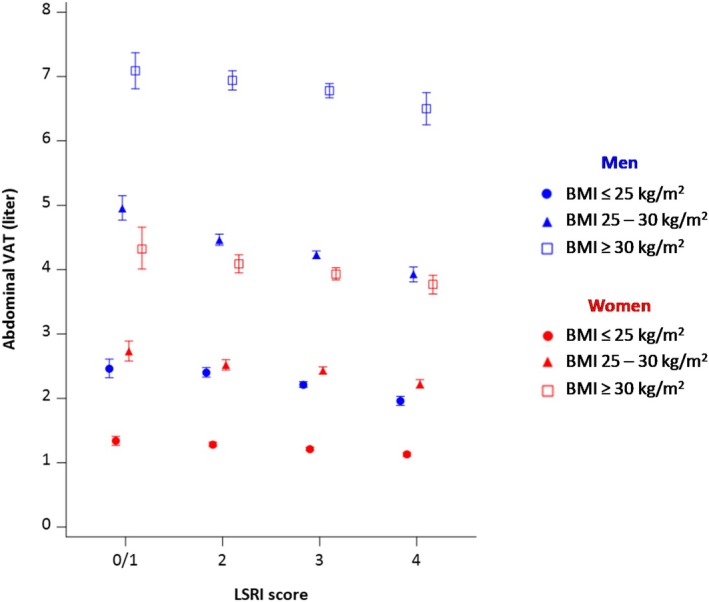
VAT volume by LSRI category and stratified by sex and BMI. Age‐adjusted geometric means ± 95% CI obtained by general linear model with abdominal visceral adipose tissue (VAT) as dependent variable and Lifestyle Risk Factor Index (LSRI) category (0/1–4 points) as independent variable stratified by sex.

In age‐adjusted analysis stratified by sex and BMI, the associations between LSRI and VAT were stronger among individuals categorized with normal weight/overweight than participants with obesity, although the associations remained statistically significant in all subgroups (Figure 3). The respective VAT levels for LSRI category 4 versus 0/1 were 20%, 21%, and 8% lower in men with normal weight, overweight, and obesity; the corresponding values for women were 16%, 19%, and 13%. This observation was also confirmed by higher regression estimates per 1‐point increase in LSRI score: −0.09, −0.07, and −0.03 for men and −0.06, −0.06, −0.04 for women across BMI categories. Similarly, the *R* ^2^ values of the full age‐adjusted models were 0.26, 0.26, and 0.18 for men and 0.23, 0.25, and 0.17 for women, progressing from normal weight to obesity (Table S4).

## 4 Discussion

In this large sample of more than 18,000 NAKO participants with information on MRI‐based body fat distribution, we observed that a higher LSRI reflecting a combination of better adherence to different healthy lifestyle behaviors was associated with lower VAT and SAT volumes. As indicated by differences in adjusted geometric means and standardized regression parameters, LSRI showed the strongest relations with VAT and the VAT/SAT ratio, whereas the differences between extreme LSRI categories were less than 5% for SAT, BMI, and WC. Among individual LSRI components, the largest differences in VAT by adherence status were detected for physical activity, followed by diet and alcohol intake, and smallest for smoking. It is noteworthy that the association of LSRI with VAT, as well as with the VAT/SAT ratio, which reflects the propensity for VAT accumulation, remained relevant after adjustment for BMI, indicating that the combination of lifestyle factors not only affects accumulation of body fat per se but also specifically the distribution within the body, that is, showing a susceptibility for VAT accumulation [8]. Stratified results further indicate that a combination of healthy lifestyle factors plays a stronger role in VAT accumulation among individuals with BMIs below 30 kg/m^2^ than those with obesity. This observation may be due to the smaller overall fat mass among persons with normal weight, which can be modified through small changes in diet or physical activity.

The prevalence of obesity and overweight in the NAKO cohort generally agrees with the distribution in the German population reported in nationwide cross‐sectional surveys [29]. A comparable report that has explored similar indices in relation to body fat distribution is based on a subset of the Multiethnic Cohort [19]. Just as in the current analysis, the same LSRI was inversely associated with the VAT/SAT ratio (*p* = 0.0004) with an estimated odds for the highest versus lowest LSRI value of 0.59 (95% CI 0.35, 0.97). Interestingly, the prevalence of the lifestyle factors was similar in the Multiethnic Cohort as in the current analysis: meeting physical activity requirement (96%), low‐to‐moderate alcohol consumption (89%), not smoking (61%), and only 22% adhering to dietary recommendations. Several other investigations examined different risk factors simultaneously. For example, an increasing number of healthy lifestyle factors was associated with lower SAT and VAT volumes in the Framingham Heart Study [23]. Based on VAT measures by computed tomography (CT) in the Insulin Resistance and Atherosclerosis Family Study, soluble fiber intake, total energy intake, and participation in vigorous activity were inversely related to VAT volume independent of change in BMI, but smoking was only related to SAT and not VAT [30].

As to individual LSRI components, substantial evidence supports the idea that physical activity is instrumental in controlling VAT accumulation. A systematic review suggests that aerobic exercise is central for programs aimed at reducing VAT [31]. In the Framingham Third Generation and Omni II cohorts with CT‐based measures, those who reported higher moderate‐to‐vigorous physical activity had lower SAT and VAT volumes (*p* < 0.0001), but the association was attenuated upon adjustment for BMI [32]. Moderate‐to‐vigorous physical activity was inversely related to abdominal adiposity in a South African study [33] and to VAT in a Brazilian study [34]. Two studies in Japan [17, 18] detected beneficial associations with VAT accumulation, while another one did not observe an association [35]. The beneficial effect of physical activity was also shown in a randomized trial showing reduced body fat and VAT [36].

There is substantial evidence that dietary factors are important in visceral fat accumulation. In a recent systematic review of 35 studies, individuals consuming a high‐quality diet, as assessed by a priori diet quality patterns based on dietary recommendations, accumulated less VAT [14]. To highlight a few reports, four a priori defined diet quality indices [37] as well as the Dietary Inflammatory Index [38] showed a strong inverse association with the VAT/SAT ratio after adjustment for total body fat in the Multiethnic Cohort. In a Dutch cohort, a 10‐point higher Dutch Healthy Diet‐index score was associated with a 2.3 cm^2^ smaller VAT area (95% CI −3.5; −1.0 cm^2^) [39].

In contrast to the weak association of alcohol intake with VAT and SAT in the present study, two Japanese studies reported that habitual alcohol drinking, together with high energy intake, was associated with a disproportionate accumulation of VAT [15], and greater alcohol consumption was associated with higher abdominal VAT area and the VAT/SAT ratio (all *p*_trend_ < 0.001), which remained significant after BMI adjustment [16]. In the Multi‐Ethnic Study of Atherosclerosis, heavy drinking versus lifetime abstention was associated with higher VAT but lower SAT [40, 41]. In the futusssre, it would be of interest to look at potential differences by type of alcohol consumed, for example, beer versus wine.

A lower BMI and SAT have been described repeatedly among smokers [13, 42], possibly due to nicotine's effect on appetite leading to eating and dietary behaviors consistent with lower energy intake [43] or a higher metabolic rate in smokers through nicotine [44]. However, limited evidence links smoking to VAT. In one study, a body composition subphenotype with unfavorable fat distribution was probably due to smoking [45]. Current smokers had higher VAT mass and VAT% than non‐smokers in a study of European and African Americans [46]. A CT‐based analysis among participants of the Coronary Artery Risk Development in Young Adults study showed that the strong association of smoking with intermuscular adipose tissue, an ectopic adipose depot associated with cardiovascular disease, was strongly attenuated by BMI [13]. A cross‐sectional study in perimenopausal women reported no significant difference in VAT between smokers and non‐smokers after adjustment for the Mediterranean Diet Score and physical activity [47].

It is clear that lifestyle factors are not solely responsible for body fat distribution. In addition to the biologic determinants of age and sex [12], it seems likely that genetic factors play a role, as indicated by the propensity of Asian populations to accumulate VAT and the relatively low proportion of VAT as compared to SAT among individuals of African American ancestry [8]. Sex hormones and aging, in particular as part of menopause, are additional determinants of body fat accumulation [48].

The current report has some strengths, foremost the very large population with MRI‐based body fat assessments using identical MRI devices, a standardized protocol, and the same operation procedures across the five imaging sites [49]. The detailed data collection of numerous lifestyle factors allowed the construction of the LSRI. However, the cross‐sectional study design did not permit causal inference over time because the risk factors and adiposity status were assessed at the same time, that is, baseline. Diet assessment was limited to an FFQ, which not only suffers from well‐known measurement errors [50], but its categories were not always able to match the food groups of the LSRI; for example, 100% fruit juice could not be distinguished from sugar‐sweetened juices. In addition, the FFQ did not allow detailed nutritional analysis, that is, the association of individual nutritional components with adiposity measurements, although the overall purpose of this study was to evaluate a combined score of lifestyle behaviors. The large proportion of participants lacking dietary information (28%) may have affected the accuracy of results, as the participants with missing dietary information had a higher BMI. Additional limitations include the likely overestimation of physical activity by GPAQ [28], the method used for this study, as more detailed physical activity measures based on accelerometry are only available for a subset of the NAKO cohort. Finally, the dichotomization of lifestyle factors probably led to a loss of information for all LSRI components. As VAT is based on the two‐point Dixon sequence, only conclusions about the volume of fat depots can be drawn. To assess the accumulation of fat, a multipoint Dixon sequence would be required.

## 5 Conclusion

The current observations support the hypothesis that a combination of alcohol consumption, smoking status, physical activity, and dietary intake, as captured by a composite LSRI, is associated with lower VAT volume independent of BMI and the strong influence of sex and age on VAT volume. The findings of stronger associations among individuals with normal than elevated BMI, which need confirmation in prospective settings and interventions, indicate that VAT levels in persons with obesity may be primarily determined by their elevated BMI rather than lifestyle behaviors alone. The results also suggest the testable hypothesis that the adoption of a healthier lifestyle, foremost an increase in physical activity as shown in the current analysis, may reduce VAT and increase muscle mass or another component, even if BMI remains stable.

## Conflicts of Interest

The authors declare no conflicts of interest.

## Supporting information


**Data S1:** oby70071‐sup‐0001‐supinfo.docx.

## Data Availability

Access to and use of NAKO (Germand National Cohort) data and biospecimens can be obtained via an electronic application portal (https://transfer.nako.de/transfer/index). The codes that support the findings of this study are available from the corresponding author upon request.
