# Supplementary material for: Association of a Lifestyle Risk Index With Visceral and Subcutaneous Adipose Tissue in the German National Cohort (NAKO)
Source: Obesity (Silver Spring). 2025 Nov 19;34(1):246–57. doi: 10.1002/oby.70071 (PMC12724031; doi:10.1002/oby.70071)
Supplement: Supplementary file 1 — Data S1: oby70071‐sup‐0001‐supinfo.docx. [file OBY-34-246-s001.docx]

**Supplementary Table 1. Definition of a Lifestyle Risk Factor Index Adapted from:**

***Lourida I, Hannon E, Littlejohns TJ, Langa KM, Hypponen E, Kuzma E, Llewellyn DJ. Association of Lifestyle and Genetic Risk With Incidence of Dementia. JAMA 2019;322: 430-437.***

| **Lifestyle Factor (max. 4 points)** | **Definition** |
| --- | --- |
| No current smoking | No self-reported current smoking |
| Healthy diet | Adherence to at least 3 of the following 7 food group recommendations:  1. Fruits: ≥ 3 servings/day  2. Vegetables: ≥ 3 servings/day  3. Fish: ≥2 servings/week  4. Processed meats: ≤ 1 serving/week  5. Unprocessed red meats: ≤ 1.5 servings/week  6. Whole grains: ≥ 3 servings/day  7. Refined grains: ≤1.5 servings/day |
| Low-to-moderate alcohol intake | US Dietary guidelines for Americans 2015-2020: up to 1 drink/day for women and up to 2 drinks/day for men.  1 drink-equivalent described as containing 14 g of pure alcohol.  Moderate consumption: women: ≤14g/day; men: ≤28g/day |
| Physical activity | ≥150 minutes moderate physical activity per week |

**Supplementary Table 2. Geometric and Arithmetic Means of Anthropometric Values by LSRI Category^a^**

| **Measures** | **Model^b^** | **LSRI score** | | | | | | | | | | | | **p-trend^c^** |
| --- | --- | --- | --- | --- | --- | --- | --- | --- | --- | --- | --- | --- | --- | --- |
|  |  | **0/1** | | | **2** | | | **3** | | | **4** | | |  |
|  |  | **Mean** | **95% CI** | | **Mean** | **95% CI** | | **Mean** | **95% CI** | | **Mean** | **95% CI** | |  |
| **BMI, kg/m^2^** | 1 | 26.7 | 26.4 | 26.9 | 26.6 | 26.5 | 26.8 | 26.3 | 26.2 | 26.3 | 25.5 | 25.4 | 25.7 | <.0001 |
|  | 2 | 26.5 | 26.3 | 26.8 | 26.5 | 26.4 | 26.6 | 26.2 | 26.1 | 26.3 | 25.6 | 25.4 | 25.7 | <.0001 |
| **WC, cm** | 1 | 92.9 | 92.1 | 93.7 | 91.8 | 91.4 | 92.2 | 90.3 | 90.0 | 90.5 | 86.5 | 86.1 | 87.0 | <.0001 |
|  | 2 | 91.9 | 91.3 | 92.5 | 90.7 | 90.4 | 91.0 | 89.4 | 89.2 | 89.6 | 87.4 | 87.0 | 87.8 | <.0001 |
|  | 3 | 91.2 | 90.9 | 91.5 | 90.1 | 90.0 | 90.3 | 89.5 | 89.4 | 89.6 | 88.9 | 88.8 | 89.1 | <.0001 |
| **Abdominal VAT, liter** | 1 | 3.2 | 3.1 | 3.3 | 3.0 | 2.9 | 3.1 | 2.7 | 2.7 | 2.8 | 2.1 | 2.1 | 2.2 | <.0001 |
|  | 2 | 3.0 | 2.9 | 3.1 | 2.8 | 2.8 | 2.8 | 2.6 | 2.5 | 2.6 | 2.3 | 2.2 | 2.3 | <.0001 |
|  | 3 | 2.9 | 2.8 | 3.0 | 2.7 | 2.7 | 2.8 | 2.6 | 2.6 | 2.6 | 2.4 | 2.4 | 2.4 | <.0001 |
| **Abdominal SAT, liter** | 1 | 6.1 | 5.9 | 6.2 | 6.0 | 6.0 | 6.1 | 5.7 | 5.7 | 5.8 | 5.5 | 5.4 | 5.6 | <.0001 |
|  | 2 | 6.2 | 6.0 | 6.4 | 6.2 | 6.1 | 6.2 | 5.9 | 5.8 | 5.9 | 5.4 | 5.3 | 5.5 | <.0001 |
|  | 3 | 6.0 | 5.9 | 6.1 | 6.0 | 6.0 | 6.0 | 5.9 | 5.9 | 5.9 | 5.7 | 5.7 | 5.8 | <.0001 |
| **Abdominal VAT/SAT ratio** | 1 | 0.50 | 0.49 | 0.51 | 0.50 | 0.49 | 0.51 | 0.47 | 0.47 | 0.48 | 0.39 | 0.38 | 0.39 | <.0001 |
|  | 2 | 0.48 | 0.47 | 0.49 | 0.45 | 0.45 | 0.46 | 0.44 | 0.44 | 0.44 | 0.42 | 0.41 | 0.42 | <.0001 |
|  | 3 | 0.48 | 0.48 | 0.49 | 0.45 | 0.45 | 0.46 | 0.44 | 0.44 | 0.44 | 0.42 | 0.41 | 0.42 | <.0001 |

^a^Abbreviations: LSRI = Lifestyle Risk Factor Index; BMI = Body Mass Index; WC = Waist Circumference; VAT = Visceral Adipose Tissue; SAT = Subcutaneous Adipose Tissue

^b^Adjusted and unadjusted geometric or arithmetic means ± 95% Confidence Intervals (95% CI) obtained by general linear model with anthropometric measurement as dependent variable and LSRI category (0/1 – 4 points) as independent variable with different covariates. For VAT, SAT and VAT/SAT ratio, the geometric mean was determined.

Model 1: unadjusted; Model 2: + sex, age; Model 3: Model 2 + BMI.

^c^p-trend obtained by general linear model with anthropometric measurement as dependent variable and continuous LSRI score as independent variable with the same covariates as above.

**Supplementary Table 3: Association of the LSRI with Standardized Anthropometric Variables^a^**

| **Measurement** | **Model 1^b^** | | | | **Model 2^b^** | | | | | **Model 3^b^** | | | | |
| --- | --- | --- | --- | --- | --- | --- | --- | --- | --- | --- | --- | --- | --- | --- |
|  | **β_st_** | **95% CI** | | **p-trend** | **β_st_** | **95% CI** | | **p-trend** | **β_st_** | | **95% CI** | | **p-trend** |  |
| **BMI (per SD)** | -0.10 | -0.11 | -0.08 | <.0001 | -0.08 | -0.10 | -0.06 | <.0001 | . | | . | . | . |  |
| **WC (per SD)** | -0.16 | -0.18 | -0.15 | <.0001 | -0.11 | -0.13 | -0.10 | <.0001 | -0.05 | | -0.06 | -0.04 | <.0001 |  |
| **Abdominal VAT, log (per SD)** | -0.20 | -0.21 | -0.18 | <.0001 | -0.13 | -0.15 | -0.12 | <.0001 | -0.09 | | -0.10 | -0.08 | <.0001 |  |
| **Abdominal SAT, log (per SD)** | -0.07 | -0.09 | -0.06 | <.0001 | -0.10 | -0.12 | -0.08 | <.0001 | -0.03 | | -0.04 | -0.02 | <.0001 |  |
| **Abdominal VAT/SAT ratio, log (per SD)** | -0.17 | -0.19 | -0.16 | <.0001 | -0.07 | -0.08 | -0.06 | <.0001 | -0.08 | | -0.09 | -0.07 | <.0001 |  |

^a^Abbreviations: LSRI = Lifestyle Risk Factor Index; BMI = Body Mass Index; WC = Waist Circumference; VAT = Visceral Adipose Tissue; SAT = Subcutaneous Adipose Tissue

**^b^**Standardized estimates ± 95% Confidence Intervals (95% CI) for LSRI with continuous LSRI (0/1-4 points) as p-trend obtained by general liner model with anthropometric measurement as dependent variable and LSRI continuous as independent variable. Model 1: unadjusted, Model 2: + sex, age; Model 3: Model 2 + BMI.

**Supplementary Table 4: Association of the LSRI with VAT by Sex and BMI Status^a^**

| **Men** | | | | | | | | **Women** | | | | | | |
| --- | --- | --- | --- | --- | --- | --- | --- | --- | --- | --- | --- | --- | --- | --- |
| **LSRI Score** | **β** | **95% CI** | | **p-value** | **p-trend** | **R^2^** | **β** | | **95% CI** | | **P-value** | **p-trend** | **R^2^** |  |
| **BMI ≤25 kg/m^2^** | | | | | | | | | | | | | | |
| **Continuous^b^** | -0.09 | -0.10 | -0.07 |  | <0.0001 | 0.26 | -0.06 | | -0.08 | -0.05 |  | <0.0001 | 0.23 |  |
| **0/1** | REF |  |  |  |  |  | REF | |  |  |  |  |  |  |
| **2** | -0.03 | -0.09 | 0.04 | 0.45 |  |  | -0.04 | | -0.10 | 0.01 | 0.14 |  |  |  |
| **3** | -0.11 | -0.17 | -0.05 | 0.001 |  |  | -0.10 | | -0.15 | -0.05 | 0.0003 |  |  |  |
| **4** | -0.23 | -0.30 | -0.16 | <0.0001 |  |  | -0.17 | | -0.23 | -0.12 | <0.0001 |  |  |  |
| **BMI 25-30 kg/m^2^** | | | | | | | | | | | | | | |
| **Continuous^b^** | -0.07 | -0.08 | -0.06 |  | <0.0001 | 0.26 | -0.06 | | -0.08 | -0.05 |  | <0.0001 | 0.25 |  |
| **0/1** | REF |  |  |  |  |  | REF | |  |  |  |  |  |  |
| **2** | -0.10 | -0.15 | -0.06 | <0.0001 |  |  | -0.08 | | -0.14 | -0.01 | 0.02 |  |  |  |
| **3** | -0.16 | -0.20 | -0.12 | <0.0001 |  |  | -0.11 | | -0.18 | -0.05 | 0.0003 |  |  |  |
| **4** | -0.23 | -0.28 | -0.18 | <0.0001 |  |  | -0.20 | | -0.27 | -0.14 | <0.0001 |  |  |  |
| **BMI ≥30 kg/m^2^** | | | | | | | | | | | | | | |
| **Continuous^b^** | -0.03 | -0.04 | -0.01 |  | 0.0005 | 0.18 | -0.04 | | -0.07 | -0.02 |  | 0.0001 | 0.17 |  |
| **0/1** | REF |  |  |  |  |  | REF | |  |  |  |  |  |  |
| **2** | -0.02 | -0.07 | 0.02 | 0.35 |  |  | -0.06 | | -0.14 | 0.03 | 0.19 |  |  |  |
| **3** | -0.04 | -0.09 | 0.00 | 0.04 |  |  | -0.09 | | -0.17 | -0.01 | 0.02 |  |  |  |
| **4** | -0.09 | -0.14 | -0.03 | 0.002 |  |  | -0.14 | | -0.22 | -0.05 | 0.002 |  |  |  |

^a^Beta estimates ± 95% Confidence Intervals (95% CI) and R-squared (R^2^) obtained by general linear regression in age-adjusted models stratified by sex and BMI status with abdominal Visceral Adipose Tissue (VAT) as dependent variable and Lifestyle Risk Factor Index (LSRI) category as independent variable.

**^b^**Obtained with continuous LSRI score as independent variable in same model as described above.
